# Larvicidal and Ovicidal Effects of Methanol Extracts From Selected Ethiopian Medicinal Plants Against *Anopheles arabiensis* and *Aedes aegypti*

**DOI:** 10.1155/jotm/4047678

**Published:** 2026-02-08

**Authors:** Lensa Tesfaye, Esayas Aklilu, Ketema Tolossa, Abebe Animut

**Affiliations:** ^1^ Aklilu Lemma Institute of Health Research, Addis Ababa University, Addis Ababa, Ethiopia, aau.edu.et

**Keywords:** *Aedes aegypti*, *Anopheles arabiensis*, efficacy, Ethiopia, larvicidal, ovicidal, plant

## Abstract

**Background:**

Synthetic insecticides face challenges, such as resistance, environmental damage, and harm to nontarget species, highlighting the need for alternative methods. Medicinal plants, along with their bioactive compounds, offer a promising solution.

**Objective:**

This study investigated the efficacy of methanol extracts derived from traditionally used Ethiopian medicinal plants against *Anopheles arabiensis* and *Aedes aegypti.*

**Methods and Materials:**

Methanol extracts (80%) of the crude plant extracts were tested on the larvae and eggs of both mosquito species at concentrations ranging from 250 to 2000 ppm. Larval mortality was recorded after 24 h of exposure, while egg hatchability was assessed after 72 h.

**Results:**

*Millettia ferruginea* exhibited the highest larvicidal activity against *Anopheles arabiensis* (LC_50_ = 461.7 ppm, LC_90_ = 1746.8 ppm), achieving 90% inhibition of egg hatching at 2000 ppm. *Momordica foetida* resulted in 85% mortality in second‐instar larvae and 80% mortality in early fourth‐instar larvae of *Anopheles arabiensis* at 2000 ppm. *Securidaca longepedunculata* demonstrated 87% larval mortality and 92% egg hatching inhibition in *Aedes aegypti* at 2000 ppm. ANOVA result shows that mortality rates varied significantly across concentrations (*p* < 0.05)

**Conclusion:**

*Millettia ferruginea*, *Momordica foetida*, and *Securidaca longepedunculata* are promising botanical insecticides. Future studies should focus on isolating active compounds to ensure environmental safety and effectiveness. These findings highlight the potential of indigenous plants for insect management and underscore the importance of traditional knowledge in the development of novel insecticides.

## 1. Background

Mosquitoes are biological vectors of various infectious diseases, including malaria, dengue fever, chikungunya, yellow fever, and Zika virus. Among Anopheles species, *Anopheles arabiensis (An. arabiensis)* serves as the principal malaria vector in sub‐Saharan Africa. In contrast, *Aedes aegypti (Ae. aegypti)* is the primary vector for viral illnesses, such as dengue, chikungunya, and Walker et al. [1]. The extensive distribution of these mosquito species across Africa poses serious public health challenges. Malaria continues to be the leading cause of morbidity and mortality, while arboviral diseases are increasingly emerging as significant health threats throughout the continent [2].

Ethiopia’s diverse altitudes, temperatures, rainfall patterns, and human activity levels create optimal conditions for the proliferation of *Anopheles* and *Aedes* mosquito species. These factors collectively lead to a heightened incidence of mosquito‐borne diseases [3]. Between January 1, 2024, and October 20, 2024, Ethiopia recorded over 7.3 million malaria cases and 1157 deaths, with a case fatality rate of 0.02%, highlighting the country’s persistent public health challenges. Four regions accounted for 81% of malaria cases and 89% of malaria‐related deaths in healthcare facilities: Oromia (44% of cases; 667 deaths), Amhara (18% of cases; 56 deaths), Southwest Ethiopia (12% of cases; 250 deaths), and South Ethiopia Regional State (7% of cases; 45 deaths) [4].

Key mosquito control measures in Ethiopia include the use of insecticide‐treated nets (ITNs) and indoor residual spraying (IRS). However, prolonged reliance on these interventions has contributed to the development of insecticide resistance, posing challenges to their continued effectiveness [5]. Recent studies across various regions of Ethiopia have indicated that *An. arabiensis* has developed resistance to multiple insecticides, including dichlorodiphenyltrichloroethane (DDT), alpha‐cypermethrin, deltamethrin, etofenprox, lambda‐cyhalothrin, and permethrin [6, 7].

Additionally, *Ae. aegypti* has demonstrated resistance to bendiocarb and propoxur in some areas. A 2022 study conducted in the southern Afar Region (Awash Sebat, Awash Arba, and Werer) reported *Ae. aegypti* mortality rates of 87% and 88% following exposure to propoxur and bendiocarb, respectively, indicating potential resistance [8]. Furthermore, synthetic insecticides contribute to environmental pollution and pose risks to nontarget organisms and human health. These challenges underscore the need for alternative, eco‐friendly, and sustainable vector control strategies [9]. The scientific community has increasingly turned to plant‐based phytochemicals, oils, and extracts as safe and cost‐effective alternatives to conventional synthetic insecticides. These natural compounds offer promising solutions for effective and environmentally sustainable vector control [10]. Ethnobotanical studies have highlighted the potential of medicinal plants as sources of mosquito repellents and insecticides [11].

In Ethiopia’s traditional healthcare system, plants have long been used to treat ailments and control biting arthropods, aligning with findings from previous studies [12, 13]. These plants contain bioactive compounds, such as alkaloids, flavonoids, terpenoids, and essential oils, which can effectively target immature mosquito stages [14]. Evaluating these plants offers valuable insights into the development of phytochemicals that are both effective and environmentally sustainable [15]. This study aimed to assess the efficacy of crude methanol extracts from Ethiopian medicinal plants against the immature stages of *An. arabiensis* and *Ae. aegypti.*

## 2. Materials and Methods

### 2.1. Plant Material Collection and Processing

Plant materials, including leaves, stems, roots, and whole plants, were collected between September and October 2023 from three kebeles (Lalisa Dimtu, Kersa Dako, and Mada Jalala) within the Arjo Gudatu District, located in the Oromia Regional State of western Ethiopia. The district lies at an altitude of 1100 to 2300 m above sea level. It receives a mean annual rainfall of approximately 1400 mm, ranging between 1200 and 2000 mm, and experiences mean annual temperatures between 18°C and 32°C. According to the Central Statistics Agency (2021), the Oromo ethnic group comprises approximately 98% of the local population.

Plant parts previously documented in ethnobotanical studies were tested for efficacy [16]. Based on local community knowledge and practices, parts of the 12 most frequently cited plant species were collected and identified. Voucher samples were deposited at the National Herbarium of Addis Ababa University, Ethiopia. Each collected plant part was washed with distilled water to remove dirt and debris and air‐dried in a shaded room at room temperature for 10 days. The dried plant material was ground into a fine powder using an electric grinder and stored in airtight containers at room temperature until extraction was performed. Fifty grams of each plant powder was placed in clean flasks and soaked in 250 mL of 80% methanol (hydro‐methanolic solvent used for extraction, consisting of 80% methanol and 20% distilled water [v/v]).

The mixture was stirred and shaken on an orbital shaker at room temperature for 72 h. Following maceration, the mixture was filtered through a Whatman No. 1 filter paper to eliminate debris. The filtrate was centrifuged at 1500 rpm for five minutes. The supernatant was transferred to a clean round‐bottom flask and concentrated using a rotary evaporator under reduced pressure at 60°C to remove the organic solvent. Any residual aqueous solvent was eliminated in an oven set at 40°C to obtain dry crude extracts. The dried extracts were weighed and stored in a refrigerator at 4°C until use in bioassays.

### 2.2. Mosquitoes Used for Testing

The *Ae. aegypti* and *An. arabiensis* larvae and ova used in this investigation were taken from laboratory colonies kept at the Vector Biology and Control Research Laboratory, Aklilu Lemma Institute of Health Research, Addis Ababa University, Ethiopia. Standard WHO [17] morphological keys were used to confirm the species identification based on characteristics, such as the larvae’s palmate hairs, comb scale arrangement, and siphon shape. The mosquito colonies were raised without the use of insecticides, with a light–dark cycle of 12:12 h, at 27°C ± 2°C and 70%–80% relative humidity. To facilitate hatching, eggs placed on damp cotton pads within adult cages were carefully moved to sterile trays filled with dechlorinated tap water. The larvae were fed finely powdered yeast every day while being raised in 1 L of dechlorinated water. To keep things sanitary and stop scum from growing, the water was changed every 2 days. Using a pipette, pupae were gathered and put in 60 × 60 × 60 cm emergence cages to develop into adults.

#### 2.2.1. Assessing Crude Extracts’ Larvicidal Effects

With a few adjustments, especially regarding concentration, the larvicidal activity of the methanol extracts was evaluated in accordance with WHO guidelines [17]. Stock solutions were prepared by dissolving 60 mg of the extract in 30 mL of 5% dimethyl sulfoxide (DMSO), a highly effective solvent for hydrophobic compounds [18]. Test concentrations of 250, 500, 1,000, and 2000 ppm were prepared by serial dilution. Twenty‐five second‐instar larvae of *An. arabiensis* were introduced into glass beakers containing 200 mL of distilled water mixed with 1 mL of the test solution. The negative controls consisted of larvae exposed to distilled water containing 5% DMSO, and each test was conducted in four replicates. Mortality was assessed 24 h post‐treatment. Similar procedures were conducted for the fourth‐instar larvae of *An. arabiensis* and *Ae. aegypti*. Dead larvae were identified as those that failed to respond to probing with a needle in the siphon or cervical region, whereas moribund larvae were defined as those unable to rise to the water’s surface or perform typical diving reactions when disturbed. Each concentration was tested in four replicates. Larval mortality percentages were calculated using the formula as follows [17]:

(1) larvae mortality rate=number of died larvaetotal number of larvae exposed∗100.

#### 2.2.2. Evaluating the Ovicidal Activity of Methanol Extracts

The ovicidal activity of the methanol extracts was assessed following the procedures outlined by Reegan et al. [19]. Twenty‐five freshly laid eggs (12 h old) of *Ae. aegypti* and *An. arabiensis* were introduced into test containers containing extract solutions at concentrations corresponding to the larvicidal assays (250–2000 ppm). The negative controls consisted of eggs exposed to distilled water containing 5% DMSO. The number of unhatched eggs at each concentration was recorded after a 72‐h exposure period. Four replicates per trial were performed for each concentration. Egg hatch inhibition rates (EHIR) were calculated using the formula as follows:

(2) EHIR=number of unhatched eggtotal number of eggs exposed∗100.

### 2.3. Phytochemical Screening

Qualitative phytochemical screening of the crude methanolic extracts used in our bioassays was performed following widely accepted procedures. A working solution was prepared by dissolving 5 mg of each crude extract in 50 mL of distilled water. Alkaloids were detected using Mayer’s and Dragendorff’s reagents; flavonoids were identified via the Shinoda and alkaline reagent tests; saponins were screened using the foam test; tannins and phenolics were detected with ferric chloride; and terpenoids were determined using the Salkowski reaction. All assays, including reagent blanks and appropriate positive controls, were performed in duplicate. Positive results were recorded based on characteristic visual indicators, such as color changes, precipitate formation, or foam persistence, in accordance with established phytochemical screening references [20].

### 2.4. Data Analysis

Statistical analyses were performed using SPSS software (Version 25.0; SPSS Inc., Chicago, IL, USA) and R (Version 4.0.5). Mean percentage mortality and standard deviation were calculated from replicate averages. Probit analysis was conducted in R to estimate lethal concentration values (LC_50_ and LC_90_), along with their 95% confidence intervals. The chi‐square (*χ* ^2^) test evaluated the goodness of fit of the probit models. Variations in egg inhibition and larval mortality across concentrations were assessed by one‐way analysis of variance (ANOVA). When significant differences were detected (*p* < 0.05), Tukey’s honest significant difference (HSD) test was applied for post hoc pairwise comparisons to identify specific group differences. Concentration groups were assigned letters (A, B, C, etc.) based on mean differences; groups sharing the same letter were not significantly different, whereas those with different letters were significantly distinct. For instance, concentration in Group A exhibited the lowest mortality rates, Group B showed intermediate rates differing statistically from Groups A and C, and Group C included with the highest concentration mortality rates.

## 3. Results

Parts of 12 indigenous plants traditionally used by the Arjo Gudatu community to control biting arthropods were collected, processed, and extracted using 80% methanol (Table 1). To compare the extract yields, approximately 50 g of powdered material from each plant part was processed. The yields of crude methanol extracts varied, ranging from 1.3 g for *Echinops sphaerocephalus* (*E. sphaerocephalus*) roots to 13 g for *Securidaca longepedunculata* (*S. longepedunculata*) roots. Other notable yields included 3.3 g from *Faurea speciosa* bark powder, 3 g from *Agave sisalana* (*A. sisalana)* leaves, and 3 g from *Millettia ferruginea (M. ferruginea*) leaves.

**TABLE 1:** Plant species and extract yields from 50 g of powdered plant material using 80% methanol (Arjo Gudatu District, East Wollega, Ethiopia).

Plant species	Part used	Amount used for extraction (g)	Extract yield (g)
*Agave sisalana Perrine ex Engel*	Leaf	50	3
*Croton macrostachyus Del*	Leaf	50	2.7
*Echinops sphaerocephalus L.*	Roots	50	1.3
*Faurea speciosa Welw.*	Bark	50	3.3
*Ficus thonningii Blume*	Bark	50	2.8
*Guizotia scabra*	Leaf	50	1.4
*Justicia schimperiana (Hochst. ex Nees) T Ander*	Leaf	50	2.9
*Lannea schimperi (A Rich) Engl.*	Bark	50	2.3
*Millettia ferruginea (Hochst. ex Nees) Tander*	Leaf	50	3
*Momordica foetida Schumach*	Leaf	50	2.4
*Securidaca longepedunculata*	Root	50	13
*Terminalia schimperiana Hochst*	Bark	50	1.6

*Note:* gm: gram.

### 3.1. Efficacy of Crude Methanol Extracts of Plant Parts Against Second‐Instar Larvae of *An. arabiensis*

The methanolic leaf extract of *M. ferruginea* induced 91 ± 0.96% mortality in second‐instar *An. arabiensis* larvae at 2000 ppm and 81% ± 1.2% mortality at 1000 ppm (Table 2). Probit analysis estimated LC_50_ and LC_90_ values of 461 ppm (95% CI: 311.9–615.2) and 1746 ppm (95% CI: 1178.9–3908.3), respectively. One‐way ANOVA revealed a significant effect of concentration on larval mortality (*F* (3, 12)= 65.23, *p* < 0.001). Tukey’s post hoc test grouped mortality at 2000 and 1000 ppm in Group C (highest mortality), 500 ppm in Group B, and 250 ppm in Group A (lowest mortality).

**TABLE 2:** Larvicidal efficacy of crude methanol extracts of plant parts from Arjo Gudatu District, East Wollega, Ethiopia, against second‐instar larvae of *An. arabiensis*.

The plant part extracted with methanol	Concentration (ppm)	% of mortality/(SD)	LC_50 (ppm) 95% CI_	LC_90 (ppm)_ (95% CI)	*X* ^2^, df = 2	ANOVA *F* (df between, df within)	*p* value	Tukey’s grouping
*Agave sisalana*	2000	71 ± 1.5	1365 (1099.64–1823.94)	3220 (2268.9–6574.4)	1.44	*F* (3, 12) = 100.568	0.001	C
	1000	34 ± 2.4						B
	500	5 ± 1.5						A
	250	0						A

*Croton macrostachyus*	2000	44 ± 0.8	2532 (1787.6–6640.6)	7255 (3733–68395.5)	2.55	*F* (3, 12) = 89.65	0.001	B
								A
	1000	5 ± 1.3						
	500	4 ± 1.4						A
	250	1 ± 0						A

*Echinops sphaerocephalus*	2000	5 ± 0.5	N/A	N/A	N/A	*F* (3, 12) = 5.6	0.12	B
	1000	1 ± 0.5						A
	500	2 ± 0.57						A
	250	0						A

*Faurea speciosa*	2000	5 ± 0.9	N/A	N/A	N/A	*F* (3, 12) = 2.063	0.16	A
	1000	4 ± 0.8						A
	500	2 ± 0.5						A
	250	0						A

*Ficus thonningii*	2000	20 ± 0.82	7380 (2736.5–5.7 × 10^8^)	54,703 (8486.6–4.08 × 10^14^)	0.16	*F* (3, 12) = 24.7	0.001	D
	1000	11 ± 1.2						C
	500	3 ± 0.5						B
	250	3 ± 0.5						B

*Guizotia scabra*	2000	2 ± 0.5	N/A	N/A	N/A	*F* (3, 12) = 1.00	0.42	A
	1000	0						A
	500	0						A
	250	0						

*Justicia schimperiana*	2000	9 ± 1.2	N/A	N/A	N/A	*F* (3, 12) = 4.17	0.31	B
	1000	3 ± 0.5						A
	500	3 ± 0.5						A
	250	2 ± 0.5						A

*Lannea schimperi*	2000	3 ± 0.96	N/A	N/A	N/A	*F* (3, 12) = 1.08	0.39	A
	1000	4 ± 0.8						A
	500	1 ± 0.5						A
	250	1 ± 0.5						A

*Millettia ferruginea*	2000	91 ± 0.96	461 (311.9–615.2)	1746 (1178.9–3908.3)	0.4	*F* (3, 12) = 65.23	0.001	C
								C
	1000	81 ± 1.2						
	500	49 ± 2.6						B
	250	29 ± 1.7						A

*Momordica foetida*	2000	85 ± 1.2	676 (535.1–850.6)	1757.9 (1299.4–2979.9)	5.07	*F* (3, 12) = 174.43	0.001	C
	1000	83 ± 1.9						C
	500	37 ± 1.7						B
	250	4 ± 0.8						A

*Securidaca longepedunculata*	2000	80 ± 0.81	542 (352.4–758.4)	2665 (1594.6–8936.8)	0.43	*F* (3, 12) = 134.3	0.001	C
	1000	76 ± 0.81						C
	500	51 ± 1.7						B
	250	23 ± 0.96						A

*Terminalia schimperiana*	2000	5 ± 1.2	N/A	N/A	N/A	*F* (3, 12) = 2.067	0.16	A
	1000	6 ± 0.58						A
	500	5 ± 1.2						A
	250	1 ± 0.5						A

Negative control (5% DMSO)	2000	0	N/A	N/A	N/A			
	1000	0						
	500	0						
	250	0						

*Note:* N/A: not applicable (the mortality rates observed at some plant concentrations were too low; it might not be possible to calculate the LC_50_ or LC_90_ values); *X* ^2^: chi‐square statistic for goodness of fit; *F* (df_1_, df_2_) = *F* value from one‐way ANOVA; df_1_ = degrees of freedom between groups; df_2_ = degrees of freedom within groups; letters explain the Tukey grouping interpretation explicitly, for instance: Means sharing the same letter are not significantly different at the 0.05 level according to Tukey’s HSD post hoc test.

Abbreviations: df, degree of freedom; Lc, lethal concentration; PPM, part per million; SD, standard deviation.

Similarly, the methanolic leaf extract of *M. foetida* caused 85% ± 1.2% mortality at 2000 ppm and 83% ± 1.9% at 1000 ppm. The LC_50_ and LC_90_ values were 676 ppm (95% CI: 535.1–850.6) and 1758 ppm (95% CI: 1299.4–2979.9), respectively. Larval mortality varied significantly across concentrations (*F* (3, 12) = 174.43, *p* < 0.001), with Tukey’s test assigning 2000 and 1000 ppm to Group C, 500 ppm to Group B, and 250 ppm to Group A.

The root extract of *S. longepedunculata* showed notable larvicidal activity, causing 80 ± 0.81% mortality at 2000 ppm and 76 ± 0.81% at 1000 ppm. The LC_50_ and LC_90_ values were 542 ppm (95% CI: 352.4–758.4) and 2665 ppm (95% CI: 1594.6–8936.8), respectively. Significant concentration‐dependent effects were confirmed (*F* (3, 12) = 134.3, *p* < 0.001), with the highest concentration grouped in Group C, 500 ppm in Group B, and lower concentrations in Group A. No mortality was observed in the negative control (5% DMSO).

### 3.2. Efficacy of Crude Methanol Extracts of Plant Parts Against Fourth‐Instar Larvae of *An. arabiensis*

The methanolic extracts showed differential larvicidal activity against fourth‐instar *An. arabiensis* larvae (Table 3). Among the tested extracts, *M. ferruginea* leaf extract exhibited the strongest activity, causing 86% ± 1.3% mortality at 2000 ppm and 85% ± 0.9% at 1000 ppm. Probit analysis estimated LC_50_ and LC_90_ values of 279 ppm (95% CI: 94.72–431.9) and 1822 ppm (95% CI: 1094.7–4319.3), respectively. One‐way ANOVA indicated significant differences among concentrations (*F* (3, 12) = 75.8, *p* < 0.001), and Tukey’s post hoc test classified the 500 ppm and 250 ppm treatments as B and A, corresponding to moderate and low mortality, respectively.

**TABLE 3:** Larvicidal efficacy of crude methanol extracts of plant parts from Arjo Gudatu District, East Wollega, Ethiopia, against fourth‐instar larvae of *Anopheles arabiensis*.

The plant part extracted with methanol	Concentration (ppm)	% of mortality/(SD)	LC_50 (ppm) 95% CI_	LC_90 (ppm)_ 95% CI	*X* ^2^, df = 2	ANOVA *F* (df between, df within)	*p* value	Tukey’s grouping
*Agave sisalana*	2000	69 ± 1.5	1399 (1093.17–2007.71)	3898 2530.98, 9763.05	1.67	*F* (3, 12) = 100.56	0.001	C
	1000	34 ± 2.4						B
	500	5 ± 1.2						A
	250	4 ± 0.5						A

*Croton macrostachyus*	2000	21 ± 1.5	7996 (2832.33–2.55 × 10^10^)	59,147 8614.62, 4.97 × 10^17^	0.473	*F* (3, 12) = 12.87	0.001	B
	1000	6 ± 1.3						A
	500	4 ± 1.4						A
	250	2 ± 0						A

*Echinops sphaerocephalus*	2000	4 ± 0.8		N/A	N/A	*F* (3, 12) = 1.00	0.4	A
	1000	4 ± 0.8						A
	500	4 ± 0.8						
	A							
	250	1 ± 0						A

*Faurea speciosa*	2000	3.06 ± 0.9		N/A	N/A	*F* (3, 12) = 3.5	0.51	C
	1000	3.06 ± 1.2						C
	500	2.04 ± 0.8						B
	250	0						A

*Ficus thonningii*	2000	34 ± 1.3	2966 1968.54, 12,326.12	8974.5 4120.37, 215,222.83	0.95	*F* (3, 12) = 65.13	0.001	B
	1000	9 ± 1.2						A
	500	1 ± 0						
	A							
	250	1 ± 0						A

*Guizotia scabra*	2000	0	N/A	N/A	N/A	*F* (3, 12) = 1.00	0.42	A
	1000	0						A
	500	0						A
	250	0						A

*Justicia schimperiana*	2000	6 ± 1.3		N/A	N/A	*F* (3, 12) = 0.57	0.64	B
	1000	6 ± 1.3						B
	500	10 ± 1.3						A
	250	1 ± 0						A

*Lannea schimperi*	2000	32 ± 4.2	5312 2262.98, 1,481,189.11	46,495 8236.44, 1.31 × 10^10^	0.83	*F* (3, 12) = 6.12	0.001	B
	1000	11 ± 1.2						A
	500	8 ± 1.8						A
								A
	250	5 ± 1.2						

*Millettia ferruginea*	2000	86 ± 1.3	279 94.72, 431.9	1822 94.72, 431.93	2.71	*F* (3, 12) = 75.8	0.001	C
	1000	85 ± 0.9						C
	500	75 ± 1.2						B
	250	39 ± 1.5						A

*Momordica foetida*	2000	85 ± 1.2	676 535.15, 850.65	1757 1299.4, 2979.9	5.07	*F* (3, 12) = 174.429	0.001	C
	1000	83 ± 1.9						
	C							
	500	37 ± 1.7						B
	250	4 ± 0.8						A

*Securidaca longepedunculata*	2000	75 ± 1.2	1000.04, 1651.42	3066 2159.14, 6109.38	3.5	*F* (3, 12) = 197	0.001	C
	1000	47 ± 1.7						B
	500	2 ± 0.57						A
								A
	250	3 ± 0.8						

*Terminalia schimperiana*	2000	6 ± 1.3	N/A	N/A		*F* (3, 12) = 3.5	0.51	C
	1000	6 ± 1.3						C
								A
	500	10 ± 1.3						
	250	1 ± 0						B

Negative control (5% DMSO)	2000							
	1000							
	500							
	250							

*Note:* N/A: not applicable (the mortality rates observed at some plant concentrations were too low; it might not be possible to calculate the LC_50_ or LC_90_ values); *χ* ^2^ (df): chi‐square goodness‐of‐fit test (with degrees of freedom); *F* (df_1_, df_2_) = *F* value from one‐way ANOVA; df_1_ = degrees of freedom between groups; df_2_ = degrees of freedom within groups; letters explain the Tukey grouping interpretation explicitly, for instance: Means sharing the same letter are not significantly different at the 0.05 level according to Tukey’s HSD post hoc test.

Abbreviations: Lc, lethal concentration; PPM, part per million; SD, standard deviation.

Similarly, the methanolic leaf extract of *M. foetida* induced 85% ± 1.2% mortality at 2000 ppm and 83% ± 1.9% at 1000 ppm, with LC_50_ and LC_90_ values of 676 ppm (95% CI: 535.2–850.7) and 1757 ppm (95% CI: 1299.4–2979.9), respectively. Mortality varied significantly across concentrations (*F* (3, 12) = 174.43, *p* < 0.001), with higher concentrations grouped in Group C, intermediate concentrations in Group B, and the lowest concentration in Group A.

The root extract of *S. longepedunculata* also showed notable larvicidal activity, causing 75% ± 1.2% mortality at 2000 ppm and 47% ± 1.7% at 1000 ppm. The LC_50_ and LC_90_ values were 1246 ppm (95% CI: 1000.0–1651.4) and 3066 ppm (95% CI: 2159.1–6109.4), respectively. Significant concentration‐dependent effects were confirmed (*F* (3, 12) = 197, *p* < 0.001), with the highest concentration classified in Group C, 1000 ppm in Group B, and lower concentrations in Group A.

Moderate larvicidal activity was observed for *A. sisalana* (69% ± 1.5% mortality at 2000 ppm; LC_50_ = 1399 ppm), *Ficus thonningii* (34% ± 1.3%; LC_50_ = 2966 ppm), and *Lannea schimperi* (32% ± 4.2%; LC_50_ = 5312 ppm), all showing significant concentration‐dependent mortality.

In contrast, extracts of *Croton macrostachyus*, *E. sphaerocephalus*, *F. speciosa*, *Guizotia scabra*, *Justicia schimperiana*, and *Terminalia schimperiana* exhibited weak or negligible larvicidal activity, with mortality rates below 25% at 2000 ppm; therefore, reliable lethal concentration values could not be determined. No mortality was observed in the negative control (5% DMSO).

### 3.3. Efficacy of Crude 80% Methanol Extracts of Plant Parts Against Second‐Instar Larvae of *Ae. aegypti*

The methanolic extracts exhibited variable larvicidal activity against *Ae. aegypti* (Table 4). The root extract of *S. longepedunculata* showed the strongest effect, achieving 87% ± 1.9% mortality at 2000 ppm and 78% ± 1.8% at 1000 ppm, with the lowest LC_50_ of 294 ppm (95% CI: 190–601) and an LC_90_ of 2095 ppm (95% CI: 1359–10,483). ANOVA revealed significant concentration‐dependent differences (*F* (3, 12) = 40.9, *p* < 0.001), with Tukey’s post hoc test grouping mortality at 2000 ppm in Group C, 1000 ppm in Group BC, 500 ppm in Group B, and 250 ppm in Group A.

**TABLE 4:** Larvicidal activity of 80% methanol extracts of plant parts from Arjo Gudatu District, East Wollega, Ethiopia, against second‐instar larvae of *Aedes aegypti*.

The plant part extracted with methanol	Concentration (ppm)	% of mortality/(SD)	LC_50 (ppm)_ (95% CI)	LC_90 (ppm)_ (95% CI)	*X* ^2^, df = 2	ANOVA *F* (df between, df within)	*p* value	Tukey’s grouping
*Agave sisalana*	2000	71 ± 1.5	1388 (1012.4 2–368.6)	5710 (3049.1–26,134.5)	1.7	*F* (3, 12) = 100.5	0.001	C
	1000	34 ± 2.3						B
	500	5 ± 1.5						A
	250	4 ± 0.5						A

*Croton macrostachyus*	2000	21 ± 1.5	8157 (2703.82–85.994)	59,788 (7331.86–5.41 × 10^12^)	0.7	*F* (3, 12) = 12.8	0.001	B
	1000	4 ± 1.2						A
	500	3 ± 1.4						A
	250	1 ± 0.5						A

*Echinops sphaerocephalus*	2000	5 ± 1.5	N/A	N/A	N/A	*F* (3, 12) = 0.6	0.63	A
	1000	4 ± 0.5						A
	500	1 ± 0.8						
	A							
	250	4 ± 0.5						A

*Faurea speciosa*	2000	5 ± 1.2	N/A	N/A	N/A	*F* (3, 12) = 1.9	0.18	A
	1000	4 ± 0.8						A
	500	2 ± 0.5						A
	250							A

*Ficus thonningii*	2000	36 ± 1.4	N/A	N/A	N/A	*F* (3, 12) = 22.5	0.001	B
	1000	10 ± 1.2						A
	500	6 ± 0.5						
	A							
	250	1 ± 0						A

*Guizotia scabra*	2000	1 ± 0.5	N/A	N/A	N/A	*F* (3, 12) = 1	0.42	A
	1000	0						A
	500	0						A
	250	0						A

*Justicia schimperiana*	2000	14 ± 0.9	N/A		N/A	*F* (3,12) = 8.4	0.001	B
	1000	5.2 ± 0.5						A
	500	4 ± 0.5						A
	250	4 ± 0.5						A

*Lannea schimperi*	2000	38 ± 0.8	2588 (1630.5–9503.8)	12,286 (4784.8–289.781)	0.7	*F* (3, 12) = 51.7	0.001	C
	1000	26 ± 0.9						B
	500	10 ± 0.5						A
								A
	250	0 ± 0						

*Millettia ferruginea*	2000	76 ± 1.7	879 (883.7–1606.1)	3573 (2297.5–8831.8)	3.0	*F* (3, 12) = 84.4	0.001	B
	1000	52 ± 2.4						B
	500	35 ± 1.5						A
	250	13 ± 0.9						A

*Momordica foetida*	2000	86 ± 1.2	713 (562.5–906)	1927 (1402–3393.9)	5.1	*F* (3, 12) = 170.5	0.001	C
	1000	83 ± 1.7						C
	500	37 ± 1.7						B
	250	4 ± 0.8						A

*Securidaca longepedunculata*	2000	87 ± 1.9	294 (190.4–601.7)	2095 (1359–10,483.2)	3.5	*F* (3, 12) = 40.9	0.001	C
	1000	78 ± 1.8						BC
	500	74 ± 1.2						B
	250	39 ± 1.3						A

*Terminalia schimperiana*	2000	10.4 ± 0.8	N/A	N/A	N/A	*F* (3, 12) = 13.4	0.001	B
	1000	4 ± 0.9						A
	500	6.25 ± 0.5						A
	250	0						A
Negative control (5% DMSO)	2000	0						
	1000	0						
	500	0						
	250	0						

*Note:* N/A: not applicable (the mortality rates observed at some plant concentrations were too low; it might not be possible to calculate the LC_50_ or LC_90_ values); *χ* ^2^ (df): chi‐square goodness‐of‐fit test (with degrees of freedom); *F* (df_1_, df_2_) = *F* value from one‐way ANOVA; df_1_ = degrees of freedom between groups; df_2_ = degrees of freedom within groups; letters explain the Tukey grouping interpretation explicitly, for instance: Means sharing the same letter are not significantly different at the 0.05 level according to Tukey’s HSD post hoc test.

Abbreviations: Lc, lethal concentration; PPM, part per million; SD, standard deviation.

The leaf extract of *M. foetida* also showed strong larvicidal potency, causing 86% ± 1.2% mortality at 2000 ppm and 83% ± 1.7% at 1000 ppm, with LC_50_ and LC_90_ values of 713 ppm (95% CI: 562–906) and 1927 ppm (95% CI: 1402–3393), respectively. Mortality varied significantly across concentrations (*F* (3, 12) = 170.5, *p* < 0.001).

The leaf extract of *M. ferruginea* exhibited 76% ± 1.7% mortality at 2000 ppm and 52% ± 2.4% at 1000 ppm, with corresponding LC_50_ and LC_90_ values of 879 ppm (95% CI: 883–1606) and 3573 ppm (95% CI: 2297–8831), respectively. ANOVA confirmed significant concentration‐dependent larvicidal effects (*F* (3, 12) = 84.4, *p* < 0.001).

Moderate larvicidal activity was observed for *A. sisalana* (71% ± 1.5% mortality at 2000 ppm; LC_50_ = 1388 ppm; LC_90_ = 5710 ppm), *L. schimperi* (38% ± 0.8% at 2000 ppm; LC_50_ = 2588 ppm; LC_90_ = 12,286 ppm), and *F. thonningii* (36 ± 1.4% at 2000 ppm).

In contrast, extracts of *C. macrostachyus* (21% ± 1.5%), *J. schimperiana* (14% ± 0.9%), *T. schimperiana* (10.4% ± 0.8%), *E. sphaerocephalus* (5% ± 1.5%), and *F. speciosa* (5% ± 1.2%) showed weak larvicidal activity, with mortality rates below 40% at 2000 ppm. Due to limited efficacy, reliable LC_50_ and LC_90_ values could not be determined. No mortality was observed in the negative control (5% DMSO).

### 3.4. Efficacy of Crude Methanol Extracts of Plant Parts Against Fourth‐Instar Larvae of *Ae. aegypti*

The methanol extracts exhibited varying larvicidal activities against mosquito larvae (Table 5). The methanol extract of *M. foetida* leaves showed the strongest larvicidal activity, causing 85% ± 1.5% mortality at 2000 ppm and 75% ± 1.8% at 1000 ppm. Probit analysis estimated LC_50_ and LC_90_ values of 691.4 ppm (95% CI: 551.3–865.5) and 1736 ppm (95% CI: 1295–2879), respectively. Mortality rates differed significantly across concentrations (*F* (3, 12) = 170.5, *p* < 0.001), with Tukey’s post hoc test grouping 2000 ppm in the highest mortality category (Group C), 1000 ppm in Group B, and lower concentrations in Group A.

**TABLE 5:** Larvicidal activity of 80% methanol extracts of plant parts from Arjo Gudatu District, East Wollega, Ethiopia, against fourth‐instar larvae of *Aedes aegypti*.

The plant part extracted with methanol	Concentration (ppm)	% of mortality/(SD)	LC_50 (ppm) 95% CI_	LC_90 (ppm)_ 95% CI	*X* ^2^, df = 2	ANOVA *F* (df between, df within)	*p* value	Tukey’s grouping
*Agave sisalana*	2000	69 ± 1.5	1388 (1012.4–2368.6)	5710 (3049.1–26,134.5)	1.7	*F* (3, 12) = 100.5	0.001	C
	1000	34 ± 2.4						B
	500	12 ± 0.5						A
	250	9 ± 1.5						A

*Croton macrostachyus*	2000	21 ± 1.5	2703.82 (85.994–862.411473.5)	3809.7 (7331.8–5.41 × 10^12^)	0.5	*F* (3, 12) = 12.8	0.001	B
	1000	5 ± 1.2						A
	500	4 ± 1.4						A
	250	2 ± 0.5						A

*Echinops sphaerocephalus*	2000	9 ± 1.5	N/A	N/A	N/A	*F* (3, 12) = 0.6	0.63	A
	1000	12 ± 0.5						A
	500	4 ± 0.9						A
	250	5 ± 0.5						A

*Faurea speciosa*	2000	4 ± 0.5	N/A	N/A	N/A	*F* (3, 12) = 13.4	0.001	A
	1000	5 ± 1.2						A
	500	4 ± 0.8						A
	250	2 ± 0.5						A

*Ficus thonningii*	2000	36 ± 1.4	3614 (2011.34–34,827.6)	18,288 (5752–2,886,583)	0.9	*F* (3, 12) = 22.5	0.001	A
	1000	10 ± 1.2						A
	500	1 ± 0						A
	250	6 ± 0.5						B

*Guizotia scabra*	2000	1 ± 0.5	N/A	N/A	N/A	*F* (3, 12) = 1	0.42	A
	1000	0						A
	500	0						A
	250	0						A

*Justicia schimperiana*	2000	14 ± 0.9	N/A	N/A	N/A	*F* (3, 12) = 8.4	0.001	B
	1000	5.2 ± 0.5						A
	500	4 ± 0.5						A
	250	4 ± 0.5						A

*Lannea schimperi*	2000	38 ± 0.8	2588 (1630.5–9503.8)	12,286 (4784.8–289.781)	0.7 3.0	*F* (3, 12) = 51.7	0.001	C
	1000	26 ± 0.9						B
	500	10 ± 0.5						A
								A
	250	0						

*Millettia ferruginea*	2000	66 ± 2.1	2588 (1630.5–9503.8)	3573 (1630.5–9503.8)	0.7	*F* (3, 12) = 84.4	0.001	B
	1000	26 ± 0.9						B
	500	10 ± 0.5						A
	250	0						A

*Momordica foetida*	2000	85 ± 1.5	691.4 (551.3–865.5)	1736 (1295–2879)	1.7	*F* (3, 12) = 170.5	0.001	C
	1000	75 ± 1.8						B
	500	37 ± 1.7						A
	250	4 ± 0.8						A

*Securidaca longepedunculata*	2000	84 ± 0.9	713 (562.5–906)	1927 (1402–3393.9)	5.1	*F* (3, 12) = 40.9	0.001	C
	1000	76 ± 1.4						BC
	500	72 ± 1.7						B
	250	29 ± 1.2						A

*Terminalia schimperiana*	2000	10.4 ± 0.8	N/A	N/A	N/A	*F* (3, 12) = 1.9	0.18	B
	1000	6.25 ± 0.5						A
	500	4 ± 0.9						A
	250	0						A

Negative control (5% DMSO)	2000	0	N/A	N/A	N/A	N/A	N/A	
	1000	0						
	500	0						
	250	0						

*Note:* N/A: not applicable (the mortality rates observed at some plant concentrations were too low; it might not be possible to calculate the LC_50_ or LC_90_ values); *χ* ^2^ (df): chi‐square goodness‐of‐fit test (with degrees of freedom); *F* (df_1_, df_2_) = *F* value from one‐way ANOVA; df_1_ = degrees of freedom between groups; df_2_ = degrees of freedom within groups; letters explain the Tukey grouping interpretation explicitly, for instance: Means sharing the same letter are not significantly different at the 0.05 level according to Tukey’s HSD post hoc test.

Abbreviations: Lc, lethal concentration; PPM, part per million; SD, standard deviation.

Similarly, the root extract of *S. longepedunculata* exhibited strong larvicidal potency, inducing 84% ± 0.9% mortality at 2000 ppm and 76% ± 1.4% at 1000 ppm. The LC_50_ and LC_90_ values were 713 ppm (95% CI: 562.5–906) and 1927 ppm (95% CI: 1402–3394), respectively. ANOVA confirmed significant variations among concentrations (*F* (3, 12) = 40.9, *p* < 0.001), with Tukey’s test categorizing 2000 ppm in Group C, 1000 ppm in Group BC, 500 ppm in Group B, and 250 ppm in Group A (Table 5).

The leaf extract of *M. ferruginea* showed moderate larvicidal activity, with 66% ± 2.1% mortality at 2000 ppm and 26% ± 0.9% at 1000 ppm. The LC_50_ and LC_90_ values were 2588 ppm (95% CI: 1630.5–9503.8) and 3573 ppm (95% CI: 1630.5–9503.8), respectively. Significant differences among concentrations were observed (*F* (3, 12) = 84.4, *p* < 0.001), with Tukey’s test grouping higher concentrations in Group B and lower concentrations in Group A.

### 3.5. Activity of 80% Methanol Extracts of Plant Parts Against the Ova of *An. arabiensis*

The methanolic extracts exhibited varying ovicidal activities against *An. arabiensis* (Table 6). The most pronounced effect was observed with *M. ferruginea* leaf extract, causing 92% ± 2.3% egg mortality at 2000 ppm and 74% ± 2.4% at 1000 ppm. This extract yielded the lowest LC_50_ value of 231 ppm and an LC_90_ of 2020 ppm, with significant differences across concentrations (*F* (3, 12) = 19.9, *p* < 0.001). Similarly, the root extract of *S. longepedunculata* also demonstrated high ovicidal potency, inducing 90% ± 2.8% mortality at 2000 ppm and 86% ± 3.3% at 1000 ppm. LC_50_ and LC_90_ values were calculated at 316 ppm and 1679 ppm, respectively (*F* (3, 12) = 11.2, *p* < 0.001).

**TABLE 6:** Ovicidal activity of 80% methanol extracts of plant parts from Arjo Gudatu District, East Wollega, Ethiopia, against ova of *Anopheles arabiensis*.

The plant part extracted with methanol	Concentration (ppm)	% of mortality/(SD)	LC_50 (ppm)_	LC_90 (ppm)_	*X* ^2^, df = 2	ANOVA *F* (df between, df within)	*p* value	Tukey’s grouping
*Agave sisalana*	2000	47 ± 2.4	N/A	N/A	N/A	*F* (3, 12) = 4.01	0.03	B
	1000	42 ± 2.4						B
	500	43 ± 2.3						B
	250	18 ± 0.8						A

*Croton macrostachyus*	2000	45 ± 0.06	N/A	N/A	N/A	*F* (3, 12) = 56.1	0.001	C
	1000	30 ± 0.08						B
	500	39 ± 0.37						A
	250	32 ± 0.9						B

*Echinops sphaerocephalus*	2000	43 ± 2.3	N/A	N/A	N/A	*F* (3, 12) = 2.9	0.07	C
	1000	42 ± 2.3						B
	500	30 ± 1.3						
	A							
	250	23 ± 2.3						A

*Faurea speciosa*	2000	61 ± 3.6	17,901	3169	2.5	*F* (3, 12) = 107.8	0.001	D
	1000	49 ± 2.2						C
	500	20 ± 0.02						B
	250	8 ± 0.02						A

*Ficus thonningii*	2000	48 ± 0.8	N/A	N/A	N/A	*F* (3, 12) = 54.7	0.001	C
	1000	37 ± 0.24						B
	500	26 ± 0.02						A
	250	10 ± 0.02						A

*Guizotia scabra*	2000	10 ± 0.5	N/A	N/A	N/A	*F* (3, 12) = 17.4	0.001	B
	1000	4 ± 0.8						A
	500	0						A
	250	0						A

*Justicia schimperiana*	2000	70 ± 1.2	1090	3169	2.5	*F* (3, 12) = 34.7	0.001	C
	1000	61 ± 1.02						AB
	500	23 ± 1.1						B
	250	18 ± 0.8						A

*Lannea schimperi*	2000	70 ± 2.5	1511	4343	0.6	*F* (3, 12) = 28.8	0.001	C
	1000	60 ± 2						AB
	500	50 ± 1.75						BC
	250	39 ± 2.2						A

*Millettia ferruginea*	2000	92 ± 2.3	231	2020	0.12	*F* (3, 12) = 19.9	0.001	C
	1000	74 ± 2.4						BC
	500	55 ± 1.8						AB
	250	41 ± 1.7						A

*Momordica foetida*	2000	77 ± 1.7	1023	2897	7.3	*F* (3, 12) = 13.7	0.001	B
	1000	74 ± 0.9						A
	500	70 ± 0.9						A
	250	50 ± 1.7						A

*Securidaca longepedunculata*	2000	90 ± 2.8	316	1679	0.49	*F* (3, 12) = 11.2	0.001	B
	1000	86 ± 3.3						B
	500	62 ± 3.6						AB
	250	41 ± 1.4						A

*Terminalia schimperiana*	2000	54 ± 0.3	2657.8	119.005	3.5	*F* (3, 12) = 18.5	0.001	C
	1000	31 ± 0.8						B
	500	30 ± 0.75						A
	250	18 ± 0.14						A

Negative control (5% DMSO)	2000	0						
	1000	0						
	500	0						
	250	0						

*Note:* N/A: not applicable (the mortality rates observed at some plant concentrations were too low; it might not be possible to calculate the LC_50_ or LC_90_ values); *χ* ^2^ (df): chi‐square goodness‐of‐fit test (with degrees of freedom); *F* (df_1_, df_2_) = *F* value from one‐way ANOVA; df_1_ = degrees of freedom between groups; df_2_ = degrees of freedom within groups; letters explain the Tukey grouping interpretation explicitly, for instance: Means sharing the same letter are not significantly different at the 0.05 level according to Tukey’s HSD post hoc test.

Abbreviations: Lc, lethal concentration; PPM, part per million; SD, standard deviation.

However, ovicidal activity was observed with *M. foetida* (77% ± 1.7% egg mortality at 2000 ppm; LC_50_ = 1023 ppm; LC_90_ = 2897 ppm), *L. schimperi* (70% ± 2.5% at 2000 ppm; LC_50_ = 1511 ppm; LC_90_ = 4343 ppm), and *J. schimperiana* (70% ± 1.2% at 2000 ppm; LC_50_ = 1090 ppm; LC_90_ = 3169 ppm).

### 3.6. Ovicidal Activity of 80% Methanol Extracts of Plant Parts From Arjo Gudatu District, East Wollega, Ethiopia, Against Ova of *Ae. aegypti*

The methanolic extracts showed varying levels of ovicidal activity against *Ae. aegypti* (Table 7). The most potent response was observed with *S. longepedunculata* root extract, which caused 92% ± 0.8% egg mortality at 2000 ppm and maintained 70% ± 1.3% mortality even at 250 ppm. This extract yielded the lowest LC_50_ value of 71 ppm and an LC_90_ of 1455 ppm, with significant differences across concentrations (*F* (3, 12) = 40.9, *p* < 0.001), whereas *M. foetida* leaf extract was similarly effective, inducing 89% ± 1.7% mortality at 2000 ppm and 79% ± 1.5% at 250 ppm. LC_50_ and LC_90_ values were calculated at 196 ppm and 2174 ppm, respectively (*F* (3, 12) = 170.5, *p* < 0.001). *M. ferruginea* also showed strong ovicidal activity, with 86% ± 1.9% mortality at 2000 ppm and 70% ± 1.3% at 250 ppm. Its LC_50_ and LC_90_ values were 377 ppm and 2082 ppm, respectively (*F* (3, 12) = 84.4, *p* < 0.001).

**TABLE 7:** Ovicidal activity of 80% methanol extracts of plant parts from Arjo Gudatu District, East Wollega, Ethiopia, against ova of *Aedes aegypti.*

The plant part extracted with methanol	Concentration (ppm)	% of mortality/(SD)	LC_50 (ppm)_	LC_90 (ppm)_	*X* ^2^, df = 2	ANOVA *F* (df between, df within)	*p* value	Tukey’s grouping
*Agave sisalana*	2000	72 ± 1.4	2117	9314	1.02	*F* (3, 12) = 100.5	0.001	C
	1000	66 ± 2.08						B
	500	62 ± 1.29						B
	250	59 ± 1.7						A

*Croton macrostachyus*	2000	28 ± 1.8	N/A	N/A	N/A	*F* (3, 12) = 12.8	0.001	C
	1000	21 ± 0.95						B
	500	19 ± 0.95						B
	250	16 ± 0.8						A

*Echinops sphaerocephalus*	2000	52 ± 0.8	2426	26,140	1.5	*F* (3, 12) = 0.58	0.63	C
	1000	22 ± 1.29						B
	500	21 ± 0.95						
	B							
	250	13 ± 1.25						B

*Faurea speciosa*	2000	52 ± 0.95	2117	9314	1.02	*F* (3, 12) = 13.4	0.001	C
	1000	41 ± 0.95						B
	500	26 ± 1.29						B
	250	10 ± 0.5						B

*Ficus thonningii*	2000	40 ± 1.8	N/A	N/A	N/A	*F* (3, 12) = 22.5	0.001	C
	1000	30 ± 1.29						B
	500	27 ± 0.9						B
	250	12 ± 0.02						A

*Guizotia scabra*	2000	13 ± 1.25	N/A	N/A	N/A	*F* (3, 12) = 1	0.42	C
	1000	12 ± 0.8						B
	500	2 ± 0.57						B
	250	0						B

*Justicia schimperiana*	2000	49 ± 1.7	N/A	N/A	N/A	*F* (3, 12) = 8.4	0.001	C
	1000	38 ± 1.29						B
	500	32 ± 0.8						A
	250	22 ± 1.29						A

*Lannea schimperi*	2000	58 ± 2.08				*F* (3, 12) = 51.7	0.001	C
	1000	51 ± 0.95						B
	500	40 ± 0.8						B
								B
	250	35 ± 1.5						

*Millettia ferruginea*	2000	86 ± 1.9	377	2082	0.5	*F* (3, 12) = 84.4	0.001	C
	1000	83 ± 0.95						C
	500	80 ± 1.6						C
	250	70 ± 1.29						B

*Momordica foetida*	2000	89 ± 1.7	196	2174	0.44	*F* (3, 12) = 170.5	0.001	C
	1000	84 ± 1.7						B
	500	81 ± 0.95						B
	250	79 ± 1.5						A

*Securidaca longepedunculata*	2000	92 ± 0.8	71	1455	0.004	*F* (3, 12) = 40.9	0.001	C
	1000	87 ± 1.5						C
	500	80 ± 0.8						B
	250	70 ± 1.29						B

*Terminalia schimperiana*	2000	47 ± 2.5	N/A	N/A	N/A	*F* (3, 12) = 1.9	0.18	C
	1000	34 ± 1.29						B
	500	20 ± 0.8						B
	250	21 ± 1.25						B

Negative control (5% DMSO)	2000	0		N/A				
	1000	0						
	500	0						
	250	0						

*Note:* N/A: not applicable (the mortality rates observed at some plant concentrations were too low; it might not be possible to calculate the LC_50_ or LC_90_ values); *χ* ^2^ (df): chi‐square goodness‐of‐fit test (with degrees of freedom); *F* (df_1_, df_2_) = *F* value from one‐way ANOVA; df_1_ = degrees of freedom between groups; df_2_ = degrees of freedom within groups; letters explain the Tukey grouping interpretation explicitly, for instance: Means sharing the same letter are not significantly different at the 0.05 level according to Tukey’s HSD post hoc test.

Abbreviations: Lc, lethal concentration; PPM, part per million; SD, standard deviation.

Moderate activity was observed in *A. sisalana* (72% ± 1.4% at 2000 ppm; LC_50_ = 2117 ppm; LC_90_ = 9314 ppm), *E. sphaerocephalus* (52% ± 0.8% at 2000 ppm; LC_50_ = 2426 ppm; LC_90_ = 26,140 ppm), and *F. speciosa* (52% ± 0.95% at 2000 ppm; LC_50_ = 2117 ppm; LC_90_ = 9314 ppm). Lower ovicidal effects were recorded for *F. thonningii* (40% ± 1.8%), *C. macrostachyus* (28% ± 1.8%), *G. scabra* (49% ± 1.7%), *J. schimperiana* (38% ± 1.3%), and *T. schimperiana* (47% ± 2.5%) at 2000 ppm. Due to limited efficacy, LC_50_ and LC_90_ values could not be reliably estimated for these extracts.

### 3.7. Phytochemical Screening of Biologically Active Plant Species

Qualitative phytochemical screening (Table 8) confirmed the presence of various secondary metabolites in the methanolic extracts of the studied plants. Alkaloids, flavonoids, terpenoids, tannins/phenolics, and saponins were the most consistently detected compounds across the extracts. *M. ferruginea* showed a particularly diverse phytochemical profile, with the presence of alkaloids, flavonoids, tannins/phenolics, and terpenoids. *A. sisalana* demonstrated positive reactions for alkaloids, tannins/phenolics, and terpenoids. The methanolic extract of *M. foetida* contained alkaloids, saponins, terpenoids, and tannins/phenolics, whereas *S. longepedunculata* was rich in flavonoids, saponins, tannins/phenolics, terpenoids, and alkaloids.

**TABLE 8:** Phytochemical screening of selected plant species used in mosquito control.

Plant species	Saponins	Tannins	Flavonoids	Alkaloids	Terpenoids	Phenols
*Agave sisalana*	−	+	+	+	+	+
*Millettia ferruginea*	−	+	+	+	+	+
*Momordica foetida*	+	+	+	+	+	+
*Securidaca longepedunculata*	+_	+	+	+	+	+

*Note:* (+) means positive and (−) = negative.

## 4. Discussion

Crude methanol extracts from the roots of *S. longepedunculata,* and the leaves of *M. ferruginea* and *M. foetida* show strong ovicidal and larvicidal activity against *Ae. aegypti* and *An. arabiensis* in this study. These results scientifically validate traditional mosquito control practices and highlight the potential of these plants as sources of environmentally friendly, plant‐based insecticides. Several studies have shown that, acknowledging the growing environmental concerns and the increasing resistance to synthetic chemical insecticides, the development of botanical alternatives is becoming increasingly critical [7, 21]. Plant‐derived bioinsecticides provide a safer, biodegradable alternative with multiple modes of action, thereby reducing the likelihood of resistance development. Their integration into property and incorporation into vector management frameworks could enhance mosquito control efforts while minimizing harmful ecological effects [22, 23]. Thus, these results underscore the importance of further research to optimize extraction methods and formulation strategies, thereby harnessing the full potential of botanical insecticides for public health applications.

The methanol extract of *M. ferruginea* leaves kills both the second‐ and fourth‐instar larvae of *An. arabiensis*, which also showed a dose‐dependent mortality response. The biological effectiveness of the extract was detailed by the ANOVA, which verified statistically significant differences among concentrations (*p* < 0.05). Interestingly, high larval mortality rates at moderate to high doses, 91% at 2000 ppm and 86% at 2000 ppm for second‐ and fourth‐instar larvae, respectively, indicate strong larvicidal activity, strengthening its potential as a successful control causal agent. This effect was further confirmed by probit analysis, which showed significant larval susceptibility with LC_50_ and LC_90_ values of 461.7 ppm and 1746.8 ppm, respectively.

In support of these findings, research on *M. ferruginea* seed extract has shown that, at relatively low concentrations (60 mg/L), it exhibits 100% larval mortality against both laboratory and field populations of *An. arabiensis*, exhibiting strong larvicidal activity against a variety of mosquito strains [24], while another study conducted in Northwest Ethiopia found that it was 98% effective at 100 ppm [25]. This outcome shows that both leaf and seed extracts may be useful in plant vector control and confirms the strong insecticidal properties of *M. ferruginea* plant parts. In this finding, *M. ferruginea’s* efficacy is probably associated with its rich secondary metabolite profile, which includes flavonoids and phenols, compounds well known for their larvicidal and ovicidal properties in this plant species.

*M. foetida* leaves exhibited significant larvicidal activity against *An. arabiensis* larvae in both second and fourth instars, causing 85% mortality at 2000 ppm and showing a clear concentration‐dependent effect (*F* (3,12) = 174.43, *p* < 0.001). Another study on *M. foetida* demonstrated high efficacy against *Anopheles gambiae* and *Anopheles coluzzii* larvae, reporting much lower LC_50_ values ranging from 204 to 271 ppm [26]. Variations in extraction methods, species‐specific susceptibility, and environmental or genetic factors of mosquito populations likely contribute to these differences in potency. Supporting this, another study showed species‐dependent response variability for *M. foetida*, achieving 100% larval mortality against *Anopheles stephensi* at 250 ppm [27]. The larvicidal effects are potentially attributed to phytochemical constituents, such as flavonoids and phenols [28]. Future research focusing on optimizing extraction techniques, identifying synergistic bioactive compounds, and broadening efficacy testing across multiple Anopheles species could enhance the potential of *M. foetida* as a plant‐based insecticide.

*S. longepedunculata* exhibited moderate larvicidal activity against second‐instar larvae *of An. arabiensis*, with 80% mortality observed at 2000 ppm. When tested on early fourth‐instar larvae, the extract caused a slightly lower mortality rate of 75% at the same concentration, indicating a modest reduction in efficacy against later larval stages. The calculated LC_50_ and LC_90_ values were 1246 ppm and 3066 ppm, respectively. An ANOVA confirmed significant differences in larval mortality across concentrations (ANOVA, *F* (3, 12) = 197, *p* < 0.001). These results are consistent with a study from Nigeria [29], which suggests that *S. longepedunculata* tends to be more effective against early larval stages. It also indicates that higher doses or the addition of synergists may be necessary to enhance efficacy against later instars.

Variations in the bioactive phytochemical profiles of the investigated plant extracts likely contribute to the observed differences in larvicidal efficacy. Both *M. ferruginea* and *M. foetida* exhibited potent larvicidal activity against *An. arabiensis* larvae, which may reflect species‐specific differences in secondary metabolites, such as flavonoids, alkaloids, and terpenoids. The differential susceptibility of larval instars, as demonstrated by stage‐specific mortality patterns in *S. longepedunculata*, underscores the importance of targeting the most vulnerable larval stages to optimize vector control interventions. These results highlight the need for integrated mosquito management approaches that incorporate detailed knowledge of larval biology and phytochemical variability to enhance the efficacy of plant‐based bioinsecticides and reduce reliance on synthetic chemicals.

Promising larvicidal activity was observed from the tested plant extracts against *Ae. aegypti* larvae, highlighting their potential as natural alternatives to synthetic larvicides. The effectiveness of each extract varied depending on the larval stage and dose, likely reflecting differences in their phytochemical compositions and modes of action [29]. Among the extracts tested, the methanol root extract of *S. longepedunculata* showed the greatest potential as a botanical larvicide against this important arboviral vector. For practical vector control interventions, the extract must demonstrate high mortality across multiple larval stages, as evidenced by the significant mortality rates at 2000 ppm for both second‐ and fourth‐instar larvae.

*S. longepedunculata* root extract exhibits strong larvicidal activity against *Ae. aegypti*, likely due to its distinct phytochemical profile, which includes bioactive compounds, such as saponins, alkaloids, flavonoids, and terpenoids known for their insecticidal properties. Supporting studies have shown that hydroethanolic extracts of *S. longepedunculata* roots contain key secondary metabolites, such as flavonoids, steroids, triterpenoids, saponins, and tannins. Notably, methyl salicylate was identified as the major constituent, comprising approximately 88% of the essential oil extracted from this plant [30]. In addition, compounds, such as humulene and methyl hexadecanoate, are found in related plant species [31, 32]. These phytochemicals likely contribute to the plant’s biological activities, underscoring its potential for mosquito control applications.

Overall, *S. longepedunculata* demonstrates promising potential as a safe, natural alternative to synthetic larvicides. Incorporating such botanical larvicides into integrated vector management could reduce chemical usage, mitigate resistance development, and support sustainable long‐term mosquito control initiatives.

The larvicidal potential of the methanol extract of *M. foetida* leaves against *Ae. aegypti* was demonstrated by an 85% mortality rate at 2000 ppm for both second‐ and fourth‐instar larvae. Probit analysis estimated the LC_50_ and LC_90_ values to be 691.4 ppm and 1736 ppm, respectively, and mortality differences across concentrations were statistically significant (*F* (3, 12) = 170.5, *p* = 0.001). However, the 80% methanol solvent system used in this study may not extract certain key larvicidal compounds optimally due to solvent polarity incompatibility. Supporting this, previous research showed that ethyl acetate extracts of *M. foetida* caused higher mortality at much lower concentrations (200 μg/mL) [33], indicating greater potency. Such disparities in extraction techniques and solvent polarity likely explain the reduced efficacy of the 80% methanol extracts.

The methanol leaf extract of *M. ferruginea* exhibited moderate but stage‐dependent larvicidal activity against *Ae. aegypti*. At 2000 ppm, it caused 85% mortality in second‐instar larvae but showed reduced efficacy with 66% mortality in early fourth‐instar larvae. Correspondingly, LC_50_ values increased from 879 ppm for second instars to 1142 ppm for early fourth instars, indicating diminished susceptibility as larvae develop. This progressive decline in larvicidal potency likely reflects physiological and biochemical changes occurring during mosquito larval growth [24].

Early instar mosquito larvae are generally more susceptible to phytochemicals due to their smaller body size, thinner cuticle, higher metabolic rate, and underdeveloped detoxification systems, which allow greater penetration and accumulation of toxic compounds. As larvae develop into later instars, they become less sensitive to botanical toxins because of enhanced enzymatic detoxification capacity, thicker cuticles, and increased physiological robustness. These physiological and biochemical changes reduce the efficacy of plant‐derived larvicides on older larvae [17]. These results particularly highlight how crucial it is to target early larval stages in mosquito control programs in order to maximize the effectiveness of plant‐derived larvicides. In order to effectively manage older larval populations in the field, they also draw attention to the possible necessity of higher concentrations or repeated applications of *M. ferruginea* extract.

The tested plant extracts’ ovicidal activity against *An. arabiensis* and *Ae. aegypti* eggs varied significantly, suggesting that they could be used as natural mosquito egg control agents. *S. longepedunculata* showed the strongest ovicidal effect at 2000 ppm, resulting in 90% and 92% egg unhatchability in *An. arabiensis* and *Ae. aegypti,* respectively. With 92% and 86% egg unhatchability for the two species, respectively, *M. ferruginea* demonstrated strong ovicidal activity, underscoring its capacity to interfere with egg viability, a crucial aspect of managing mosquito populations. Flavonoids, alkaloids, and tannins are among the many phytochemicals found in the roots of *S. longepedunculata* and the leaves of *M. ferruginea*. The insecticidal properties of this composition are well‐known. This is supported by studies conducted on phytochemical‐rich *M. ferruginea* extract [20].

Additionally, another study reported the presence of carbohydrates, terpenoids, cardiac glycosides, saponins, and flavonoids in the root bark of *S. longepedunculata* [34]. The growth‐regulating and insecticidal qualities of these secondary metabolites are well‐known. In particular, flavonoids and alkaloids may disrupt the balance of hormones and enzyme systems necessary for embryonic development, resulting in unsuccessful egg hatching [35, 36]. It is also known that saponins and tannins can cause embryo mortality by altering the permeability of the egg chorion, which impedes gas exchange and water regulation [37].

Extracts from *M. foetida* also demonstrated marked ovicidal potential, achieving 77% and 89% egg unhatchability in *An. arabiensis* and *Ae. aegypti*, respectively. Notably, *M. foetida* showed the lowest LC_50_ value (196.5 ppm) for *An. arabiensis*, indicating high potency even at lower concentrations. The differences in ovicidal activity between mosquito species may reflect species‐specific susceptibility to the bioactive compounds.

These differences are probably caused by varying concentrations and kinds of phytochemicals, such as phenolics, tannins, and saponins, which impair the integrity of the egg chorion and embryonic development, hence decreasing hatchability. *S. longepedunculata*, *M. ferruginea*, and *M. foetida* exhibit superior ovicidal activity, indicating that their bioactive compounds efficiently break through the protective layers of mosquito eggs or interfere with embryogenesis. These qualities highlight their potential as long‐term botanical alternatives for controlling mosquito populations.

Furthermore, research indicates that effectiveness varies depending on the type of extract and the plant species, with solvent polarity influencing both the effectiveness of plant extraction and the larvicidal/ovicidal results. Hexane extracts, for example, which are known to contain nonpolar compounds, have shown strong ovicidal potential in certain plants [38]. In future studies, the plant extraction process should be optimized by carefully selecting solvents of appropriate polarity to maximize both the yield and the bioactivity of the target compounds.

In contrast to the other tested plants, *C. macrostachyus* and *G. scabra* exhibited limited efficacy, achieving less than 50% mortality or hatching inhibition, even at the highest concentrations tested. These findings suggest that their phytochemical profiles may lack the potency or sufficient concentrations of the active compounds required for effective mosquito control. This emphasizes the variability in insecticidal potential among plant species and highlights the importance of selecting plants with robust bioactive properties for use in mosquito control.

## 5. Conclusion

This study underscores the significant potential of Ethiopian plants, particularly *S. longepedunculata* and *M. ferruginea*, as effective larvicidal and ovicidal agents against major mosquito vectors. Their utilization could complement existing insecticidal strategies by targeting critical egg and larval stages, disrupting the mosquito life cycle, and reducing the population density. These findings lay the groundwork for developing sustainable plant‐based mosquito control strategies that address the shortcomings of synthetic insecticides. Future research should prioritize the isolation and characterization of the active compounds within these promising plants, uncover their mechanisms of action, and validate their efficacy under field conditions. Such efforts could meaningfully contribute to integrated vector management programs and global initiatives aimed at combating vector‐borne diseases.

## Funding

This research did not receive any specific funding from the public, commercial, or not‐for‐profit sectors.

## Ethics Statement

This research did not involve human or animal subjects.

## Consent

The authors have nothing to report.

## Conflicts of Interest

The authors declare no conflicts of interest.

## Data Availability

The data that support the findings of this study are available in the supporting information of this article.
